# Oxytocin, Feeding, and Satiety

**DOI:** 10.3389/fendo.2013.00035

**Published:** 2013-03-20

**Authors:** Nancy Sabatier, Gareth Leng, John Menzies

**Affiliations:** ^1^Centre for Integrative Physiology, School of Biomedical Sciences, The University of EdinburghEdinburgh, UK

**Keywords:** oxytocin, food, appetite, satiety, reward

## Abstract

Oxytocin neurons have a physiological role in food intake and energy balance. Central administration of oxytocin is powerfully anorexigenic, reducing food intake and meal duration. The central mechanisms underlying this effect of oxytocin have become better understood in the past few years. Parvocellular neurons of the paraventricular nucleus project to the caudal brainstem to regulate feeding via autonomic functions including the gastrointestinal vago-vagal reflex. In contrast, magnocellular neurons of the supraoptic and paraventricular nuclei release oxytocin from their dendrites to diffuse to distant hypothalamic targets involved in satiety. The ventromedial hypothalamus, for example, expresses a high density of oxytocin receptors but does not contain detectable oxytocin nerve fibers. Magnocellular neurons represent targets for the anorexigenic neuropeptide α-melanocyte stimulating hormone. In addition to homeostatic control, oxytocin may also have a role in reward-related feeding. Evidence suggests that oxytocin can selectively suppress sugar intake and that it may have a role in limiting the intake of palatable food by inhibiting the reward pathway.

## Introduction

Unlike Roald Dahl’s “enormously fat” Augustus Gloop – a boy who pursued eating as a hobby – humans usually eat much less than they could. Obesity is typically the result of a modest excess of energy intake over expenditure, but one that is sustained over a prolonged period. In fact, humans, like other animals, are very efficient at balancing energy intake and expenditure. If rats are allowed unlimited access to a high-energy palatable diet, their energy intake diverges quickly from control animals fed a bland diet, but the difference in energy intake stabilizes within days (Archer and Mercer, 2007). Thus rats, which presumably do not feel societal pressure to be slim, will overeat palatable food, but only to a certain extent. When their access to palatable food ends, rats typically *undereat*, failing to defend the extra body weight they have accumulated.

A variety of peripheral signals convey information to control meal size and, in the longer term, these signals are modified according to physiological state and the size of energy reserves. Several “satiety” peptides are secreted from the gastrointestinal tract, including cholecystokinin (CCK), glucagon-like-peptide-1 (GLP-1), and peptide YY (PYY) (Strader and Woods, 2005); some of these act on the brain at sites that lack a blood-brain barrier, others are transported across the blood-brain barrier, and others act via vagal neuronal afferents (Verbalis et al., 1986). Leptin, which is secreted from adipose tissue in proportion to the size of fat stores, is not itself a satiety signal, but by signaling the size of peripheral fat reserves it has a long term anorectic influence, and in part it may act by moderating acute satiety signals arising from the gastrointestinal tract. Whether these satiety signals and leptin all converge at a discrete “satiety center” in the brain is unclear but several neuronal populations have been identified as likely candidates for mediating satiety. In part, these have been identified because they synthesize peptides that have marked anorexic actions when administered centrally.

One of those potently anorectic neuropeptides is oxytocin – a peptide classically thought to be involved mainly in reproductive functions. However, as we review here, there is now considerable evidence that oxytocin also plays an important role in satiety.

## Physiological Roles of Oxytocin

Oxytocin is produced in two hypothalamic regions: the supraoptic nucleus (SON) and paraventricular nucleus (PVN). Magnocellular neuroendocrine neurons in these nuclei project to the posterior pituitary gland, from where oxytocin is secreted into the blood. Classically, oxytocin secreted from the pituitary gland is involved in efficient and timely fetal expulsion, and is indispensable for the milk-ejection reflex and successful lactation (Nishimori et al., 1996); in some species, including rodents but not humans, it also regulates sodium excretion (natriuresis) (Verbalis et al., 1991), both by direct actions at the kidneys and indirectly be regulating the secretion of natriuretic peptides from the heart (McCann et al., 2003); in these species it is secreted in response to raised plasma osmotic pressure (Huang et al., 1996). All of the oxytocin neurons in the SON are magnocellular neuroendocrine neurons, but the PVN also contains parvocellular oxytocin neurons that project within the brain – to the spinal cord, caudal brainstem, amygdala, and substantia nigra (Sofroniew, 1980). These neurons are important in sexual behavior in males (Melis et al., 1986), and, as we discuss below, in the regulation of gastric reflexes.

Recently it has become apparent that oxytocin has an important “pro-social” role. In rodents, it has been implicated in recognition and positive social behavior between rodent mothers and their offspring and between adult members of the same social group (Neumann, 2009). However, behaviors linked to oxytocin are not all positive. In the rat, for example, patterns of central oxytocin release correlate with maternal aggression against unfamiliar intruders (Bosch et al., 2005). Various “social” effects of oxytocin in humans have also been reported, but mainly following the intranasal administration of doses of oxytocin so large that it is hard to be confident of where or how they are acting.

It is unclear to what extent these “behavioral” roles of oxytocin are attributable to the parvocellular oxytocin system or the magnocellular system, because many of the sites of action of oxytocin in this regard lack conspicuous innervation by oxytocin-containing fibers. Accordingly, it seems likely that oxytocin reaches these sites by volume transmission following release from possibly quite distant sites. Large amounts of oxytocin are released in some circumstances from the dendrites of magnocellular neurons (Ludwig and Leng, 2006), and interestingly, such dendritic released is regulated independently of axonal secretion – thus magnocellular neurons can release oxytocin either centrally or peripherally depending upon the stimulus (Sabatier et al., 2003).

## Oxytocin and Food Intake

In early studies of hypothalamic function, lesions of oxytocin-containing hypothalamic nuclei were shown to result in an increase in food intake and body weight (Leibowitz et al., 1981; Shor-Posner et al., 1985; Sims and Lorden, 1986; Kirchgessner et al., 1988). Then, in the 1990s, several studies reported anorexigenic effects of central oxytocin: low doses of oxytocin given icv dose-dependently inhibited food intake in rats, increased the latency to begin feeding and reduced meal duration in both hungry and satiated animals, and these actions could be blocked by oxytocin receptor antagonists (Arletti et al., 1990; Olson et al., 1991a). Longer term central infusions of oxytocin were also reported to reduce body weight gain in rats given a high-fat diet, but in contrast to oxytocin’s acute effects, chronic oxytocin infusions did not alter total food intake or meal patterning, but instead appeared to stimulate lipid metabolism in adipose tissue (Deblon et al., 2011). It was noted that, in rats, dehydration or sodium loading potently increased oxytocin secretion and at the same time suppressed appetite (Flanagan et al., 1992a); given that (in rats) oxytocin promotes natriuresis (Verbalis et al., 1991), the suppression of appetite by oxytocin appeared to be part of a general homeostatic role in sodium balance. As oxytocin secretion is not stimulated by hyperosmolarity in humans (Williams et al., 1986) it seemed that this role of oxytocin might be one peculiar to rodents. However, recent findings in humans with rare genetic mutations linked to monogenic obesity indicate that oxytocin may also have a role in appetite regulation in humans.

Male (but not female) oxytocin receptor-deficient mice express an obese phenotype in later adulthood despite no difference in food intake or motor activity (Takayanagi et al., 2008). Male and female oxytocin-knockout mice show an elevation in body weight and fat stores in adulthood but as with oxytocin receptor-deficient mice this is not due to an increase in food intake (Nishimori et al., 1996). There have been no published reports of humans completely lacking either oxytocin or its receptor, probably because the absence of oxytocin or its receptor is incompatible with successful reproduction, but a partial deficiency in central oxytocin production has been associated with the development of obesity in humans in two documented conditions.

The transcription factor Single-minded 1 (Sim1) is one of the few genes associated with human monogenic obesity (Holder et al., 2000; Farooqi and O’Rahilly, 2005). In mice, Sim1 is expressed in the SON and PVN and is essential for the development of these nuclei (Michaud et al., 1998). Homozygous Sim1 knockout mice do not survive gestation; but heterozygous mice are viable and are hyperphagic and become obese early in life (Michaud et al., 2001). These mice have much-reduced levels of oxytocin mRNA and immunoreactive oxytocin in both the SON and PVN (Michaud et al., 2001), and their hyperphagia can be reversed by oxytocin given icv (Kublaoui et al., 2008). Conditional deletion of Sim1 after gestational development of the PVN also results in both a reduction in oxytocin mRNA expression, and in hyperphagia and obesity (Tolson et al., 2010). In contrast, mice overexpressing Sim1 do not increase their food intake when given a high-fat diet, and are resistant to diet-induced obesity (Kublaoui et al., 2006b).

Patients affected by Prader–Willi syndrome (PWS) caused by the lack of a segment in the paternal chromosome 15 suffer from morbid obesity due to extreme hyperphagia. The PVN of these patients contains fewer oxytocin neurons than controls (Swaab et al., 1995), leading to speculation that a deficiency in oxytocin may be instrumental in the development of obesity in this condition. Mice in which specific genes associated with the PWS syndrome have been knocked out similarly develop late-onset obesity due to hyperphagia, and this can be partly explained by a deficient production of oxytocin in the hypothalamus (Dombret et al., 2012) or by a reduction in the number of oxytocin neurons (Muscatelli et al., 2000).

## Where Does Appetite-Inhibitory Oxytocin Come From?

In the last 20 years it has become apparent that oxytocin neurons in both the SON and PVN are powerfully regulated by appetite-related signals (Renaud et al., 1987; Olson et al., 1991a,b). The role of central oxytocin in the regulation of energy homeostasis appears to involve both the magnocellular neurons and centrally projecting parvocellular neurons.

### Parvocellular neurons of the PVN

The PVN has a major role in the regulation of appetite and metabolism, and is an important direct target of projections from the primary leptin- and ghrelin-receptive neurons of the arcuate nucleus – from orexigenic neurons that co-express neuropeptide Y (NPY) and agouti-related peptide (AgRP) and the inhibitory neurotransmitter GABA, and from pro-opiomelanocortin (POMC)-containing neurons, which express the potent satiety peptides α-melanocyte stimulating hormone (α-MSH) and cocaine-and amphetamine regulating transcript (CART) (Valassi et al., 2008). The PVN regulates metabolism via neuroendocrine neurons that release thyrotropin releasing hormone to regulate the thyroid gland (Alkemade, 2010; Nillni, 2010), it regulates glucocorticoid production via its regulation of pituitary adrenocorticotropin secretion (Herman et al., 2003), and it regulates the sympathetic nervous system via a large population of pre-autonomic neurons (Ferguson et al., 2008; Kc and Dick, 2010). However, the oxytocin neurons in the PVN, like those in the SON, are also conspicuous targets for α-MSH (Kim et al., 2000), and are particularly powerfully influenced by food intake and a variety of nutritionally related signals.

In rats, the expression of oxytocin mRNA in the PVN is markedly reduced by fasting; this reduction can be reversed by leptin administration (Kublaoui et al., 2008) and these effects apparently involve both magnocellular neurons and parvocellular neurons. Oxytocin neurons in the PVN are also contacted by fibers arising from the NPY/AgRP/GABA neurons of the arcuate nucleus. The PVN expresses abundant GABA receptors (Kalsbeek et al., 2004) and NPY Y1 receptors (Yokosuka et al., 1999). Optogenetic activation of the GABAergic axons in the PVN that arise from the arcuate nucleus increases food intake. Similarly, direct optogenetic activation of PVN oxytocin neurons increases c-Fos expression in these neurons and suppresses food intake. In the same study, a pharmacogenetic approach showed that while acute silencing of arcuate POMC neurons has surprisingly little effect, silencing Sim1-expressing PVN neurons markedly increases food intake (Atasoy et al., 2012).

Many parvocellular oxytocin neurons project to the nucleus tractus solitarii (NTS) in the caudal brainstem (Rinaman, 1998) where oxytocin modulates vagal efferent pathways that regulate gastric motility (McCann and Rogers, 1990). These neurons are critically involved in a reflex that is triggered by food intake, and mediated in part by gastric distension and in part by the secretion of CCK from the duodenum. Peripheral administration of CCK leads to activation of gastric vagal afferent neurons and thence to activation of brainstem structures, notably the NTS and ventrolateral medulla (Simpson et al., 2012). These in turn project to, and activate, centrally projecting oxytocin neurons of the PVN. As the CCK-stimulated NTS neurons are densely innervated by oxytocin-containing fibers from the PVN (Blevins et al., 2003), it appears that there is a recurrent circuit involving parvocellular oxytocin neurons that modulates the gastrointestinal vago-vagal reflex.

The gastrointestinal vago-vagal reflex involves main three-components: the gastrointestinal tract, the NTS, and the dorsal motor nucleus of the vagus (DMV). Visceral afferent fibers carrying digestion-related information ascend the vagus nerve and terminate in the medial NTS. NTS neurons integrate the information and send afferent projections to the DMV, and DMV neurons in turn project back down to the intrinsic ganglia in the gastrointestinal tract. In addition to this main loop, catecholaminergic and peptidergic neurons of the NTS also send ascending projections to structures in the forebrain including the PVN and the SON, and parvocellular PVN neurons project back to both the NTS and the DMN (Saper et al., 1976; Swanson and Kuypers, 1980). There is clear evidence that oxytocin acts in the DMN to inhibit gastric motility: the DMN contains oxytocin receptors (Dubois-Dauphin et al., 1992) and injection of oxytocin into the DMN decreases gastric motility, while oxytocin antagonists have the opposite effect (Rogers and Hermann, 1987). Electrical stimulation of the PVN inhibits gastric motility, and this effect can be attenuated by injection of an oxytocin antagonist in the DMN (Rogers and Hermann, 1987). Similar results were observed in conscious, freely moving animals, in which gastric motility was reduced by icv injection of oxytocin and by stimulation of the PVN, and in both cases the inhibition was prevented by icv injection of an oxytocin antagonist (Flanagan et al., 1992b). Finally, icv administration of an oxytocin antagonist alone increased baseline gastric motility, suggesting a tonic inhibitory effect of oxytocin on gastric motility. However, it seems unlikely that the inhibitory action of CCK on gastric motility is mediated by oxytocin, as icv injection of an oxytocin antagonist did not prevent CCK-induced inhibition of gastric motility (Flanagan et al., 1992b).

Thus there is a well-established role of parvocellular oxytocin neurons in the regulation of the gastrointestinal tract. Moreover, the magnocellular oxytocin neurons of the SON and PVN also appear to be involved in regulating appetite, possibly by the actions of dendritically released oxytocin on the ventromedial nucleus of the hypothalamus (VMN). This large nucleus is known to have an important role in both energy balance and sexual behavior, and is a site at which oxytocin receptors are expressed at an exceptionally high density (Tribollet et al., 1988) as well as insulin-regulated aminopeptidase (IRAP), an enzyme involved in the inactivation of oxytocin (Fernando et al., 2005).

### Magnocellular neurons of the SON and PVN

As we discuss further below, there is powerful evidence that magnocellular oxytocin neurons have an important role in the regulation of appetite – but it is important to note that this role is not necessarily mediated by oxytocin alone. In magnocellular neurons of the SON and PVN, oxytocin is co-localized with a number of anorexigenic factors, including the neuropeptides CART (Vrang et al., 1999), pituitary adenylate cyclase activating polypeptide (PACAP) (Koves et al., 1994), corticotropin-releasing factor (CRF) (Sawchenko et al., 1984), CCK (Vanderhaeghen et al., 1981), and nesfatin-1 (Foo et al., 2008). Indeed, it has been suggested that oxytocin actually mediates the inhibitory action of nesfatin-1 on food intake, as nesfatin-1 induces the release of oxytocin in the PVN (Maejima et al., 2009) and as the anorexigenic effect of nesfatin-1 can be blocked by an oxytocin antagonist (Yosten and Samson, 2010). The fat mass and obesity-associated (FTO) gene that has been associated with obesity in humans is also co-localized with oxytocin in both the PVN and SON. This gene encodes a transcription co-factor (Fto) that is believed to regulate the expression of appetite-related genes (Jia et al., 2008), and Fto over-expression increases oxytocin mRNA levels in cell cultures (Olszewski et al., 2011).

Magnocellular oxytocin neurons are activated during feeding: thus, in schedule-fed rats trained to expect to receive food for just 2 h each day, magnocellular oxytocin neurons in both the SON and PVN densely express Fos protein soon after the initiation of food intake (Johnstone et al., 2006). These neurons are also activated by gastric distension, and by systemic application of the satiety peptides CCK (Renaud et al., 1987) and GLP-1 (Bojanowska and Stempniak, 2000). Moreover, the intestinal lipid amide oleoylethanolamide (OEA), which suppresses feeding via activation of the vagus nerve (Lo Verme et al., 2005), stimulates oxytocin mRNA expression in the PVN and SON, this anorexic effect is prevented by blockade of central oxytocin receptors (Gaetani et al., 2010).

The best understood pathway involving feeding-evoked activation of magnocellular oxytocin neurons is that where peripheral injections of CCK leads to secretion of oxytocin from the posterior pituitary gland in rats. In brief, CCK is released from the duodenum in response to food intake and peripheral administration of CCK can inhibit food intake via stimulation of vagal afferent neurons and activation of brainstem structures, notably the NTS and ventrolateral medulla. CCK given intraperitoneally or intravenously increases Fos expression in oxytocin neurons in the PVN and SON (Caquineau et al., 2010), increases the electrical activity of oxytocin neurons in the SON (Renaud et al., 1987; Leng et al., 1991) and increases plasma oxytocin secretion (Kutlu et al., 2010). These actions are mediated by a direct projection from noradrenergic neurons of the A2 cell group, which co-express the potent appetite-inhibiting peptide prolactin-releasing peptide (PrRP). The activation of oxytocin neurons can be blocked by selective lesioning of the noradrenergic afferents or by blocking the actions at the SON of noradrenaline itself or those of PrRP (Onaka et al., 2012). Interestingly, central pretreatment with an oxytocin receptor antagonist reduces the anorexigenic effect of CCK (Olson et al., 1991b; Blevins et al., 2003) suggesting that CCK-evoked satiety may be mediated in part by oxytocin.

### Interactions between magnocellular oxytocin neurons and the melanocortins

Like parvocellular oxytocin neurons, magnocellular oxytocin neurons abundantly express leptin receptors and are a target for this important hormone (Hakansson et al., 1998; Yarnell et al., 1998; Brogan et al., 2000). Leptin activates Fos expression in the PVN (Yokosuka et al., 1998; Caquineau et al., 2010; Qi et al., 2010), particularly in parvocellular PVN neurons projecting to CCK-sensitive neurons in the NTS. In the SON, however, central administration of leptin does not induce Fos expression (Caquineau et al., 2010) or nuclear translocation of STAT3 (Hakansson and Meister, 1998), but it does increase nuclear STAT5 expression (Mutze et al., 2007).

In addition to this direct modulation by leptin, magnocellular oxytocin neurons are regulated indirectly via the effects of leptin on POMC neurons of the arcuate nucleus. Like the PVN, the SON receives a strong projection from POMC neurons. Oxytocin neurons in the SON, like those in the PVN, densely express α-MSH receptors (MC4) (Garza et al., 2008). α-MSH is a powerful anorexigenic peptide: centrally administered α-MSH reduces food intake and body weight, and mice lacking MC4 receptors are hyperphagic and obese (Adan et al., 2006). As mentioned above, magnocellular oxytocin neurons can secrete a large amount of peptide from their dendrites in response to certain stimuli, and notably they do so in response to α-MSH. In magnocellular oxytocin neurons of the SON, α-MSH acts at MC4 receptors to increase the intracellular calcium concentration; this increase directly evokes oxytocin release from the large dendrites of these neurons and also results in the production of endocannabinoids by the oxytocin neurons. Endocannabinoids produced in response to α-MSH act presynaptically to suppress glutamatergic afferent inputs to the oxytocin neurons. Thus, remarkably, the response of magnocellular oxytocin neurons to α-MSH is an increase in central release of oxytocin but a simultaneous suppression of electrical activity and hence a suppression of secretion into the systemic circulation (Sabatier and Leng, 2006).

Further evidence of an interaction between oxytocin and α-MSH is illustrated in the model of Sim1 heterozygous mice. In wild type mice, an agonist of α-MSH MC4 receptor (MC4R) reduced food intake, and induced Fos expression in PVN neurons, of which some co-express oxytocin and MC4R (Liu et al., 2003), and oxytocin and Sim1 (Kublaoui et al., 2008). However in Sim1 heterozygous mice, MC4R agonist had a much attenuated effect on both food intake and Fos expression in the PVN (Kublaoui et al., 2006a). This suggests that the α-MSH agonist actions on the PVN of Sim1^±^ mice were impaired by the lack of oxytocin production in these mice (Michaud et al., 2001).

### The ventromedial nucleus of the hypothalamus – a site of action of oxytocin

It seems possible that oxytocin released from the dendrites of magnocellular neurons is involved in the regulation of appetite, presumably reaching its targets by volume transmission. We have estimated that a release rate of just one vesicle per oxytocin cell every 10 s would be enough to achieve a mean basal concentration of ∼260 pg/ml throughout the anterior hypothalamus within a minute (Leng and Ludwig, 2008). The half life of oxytocin in CSF is ∼20 min – it is likely to be less than this in the extracellular fluid, but there are no clear data on this point. We have argued elsewhere that the effects of oxytocin depends less on its sites of release but rather on the location of its receptors (Sabatier et al., 2007). Thus it is likely that food intake is inhibited in various physiological conditions in which oxytocin is released from the dendrites of magnocellular neurons. Indeed, appetite is inhibited in rats subjected to dehydration or sodium loading (Flanagan et al., 1992a), two stimuli that result in a hyperosmolar environment, which is known to stimulate the dendritic release of oxytocin from supraoptic neurons (Neumann et al., 1993). The main physiological circumstances in which there is extensive central secretion of oxytocin is in lactation, when suckling-induced dendritic oxytocin release is an essential part of the milk-ejection reflex (Rossoni et al., 2008). Lactation is associated with a marked increase of food intake, rather than a reduction, but despite this, lactation is a time of negative energy balance, because the increased food intake does not adequately compensate for the energy demands of the suckling young. Accordingly, it seems that appetite during lactation is not increased to the degree needed to maintain energy homeostasis – and it may be that suppression of appetite during suckling is necessary to ensure that the mother nurses the young rather than searches for food.

One particularly intriguing potential target for dendritic oxytocin is the VMN. This large hypothalamic nucleus contains a very high density of oxytocin receptors, as shown by intense labeling both for oxytocin receptor binding sites (Tribollet et al., 1988) and oxytocin receptor mRNA expression (Yoshimura et al., 1993), particularly in the ventrolateral region. Oxytocin receptors in the VMN have an established role in sexual behavior in female rats (McCarthy et al., 1994), and we have previously suggested that they are involved in the reciprocal regulation of appetite and sexual behavior (Leng and Ludwig, 2008). Although the VMN has a high density of oxytocin receptors, it contains very few oxytocin fibers, and is therefore a likely target for oxytocin released from the dendrites of magnocellular oxytocin neurons (Leng and Ludwig, 2008).

**Figure 1:**
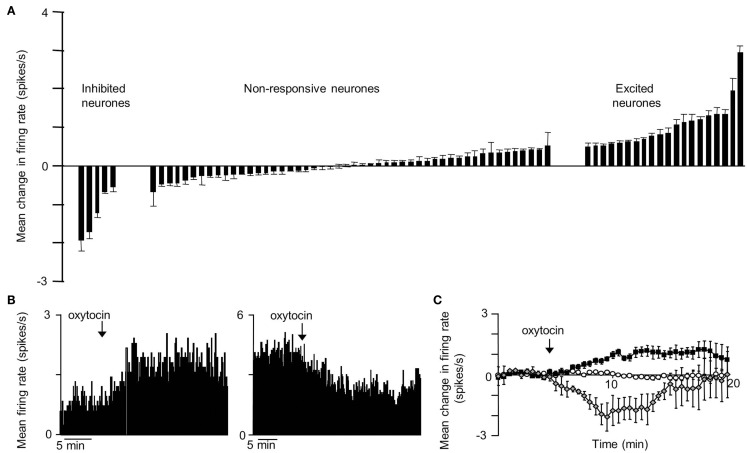
**Responses to icv oxytocin in VMN neurons *in vivo***. Single VMN neurons were recorded extracellularly from the VMN of urethane-anesthetized rats (Sabatier and Leng, 2008). **(A)** Each bar represent the mean change in firing rate (±SD) averaged over the first 10 min after icv injection of oxytocin (1–10 ng) in each VMN cell tested. The cells are ranked by response magnitude and are classed as inhibited, non-responsive, and excited according to whether the responses were significant or not. **(B)** Representative example of an inhibition (left panel) and activation (right panel) of the mean firing rate in response to icv oxytocin in a single VMN cell recorded extracellularly. **(C)** Mean change in firing rate (±SE) in the VMN cells that were significantly activated 
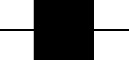
; *n* = 14), significantly inhibited 
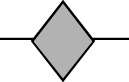
; *n* = 4), and not significantly affected 
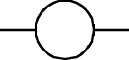
; *n* = 43) by icv oxytocin.

**Figure 2:**
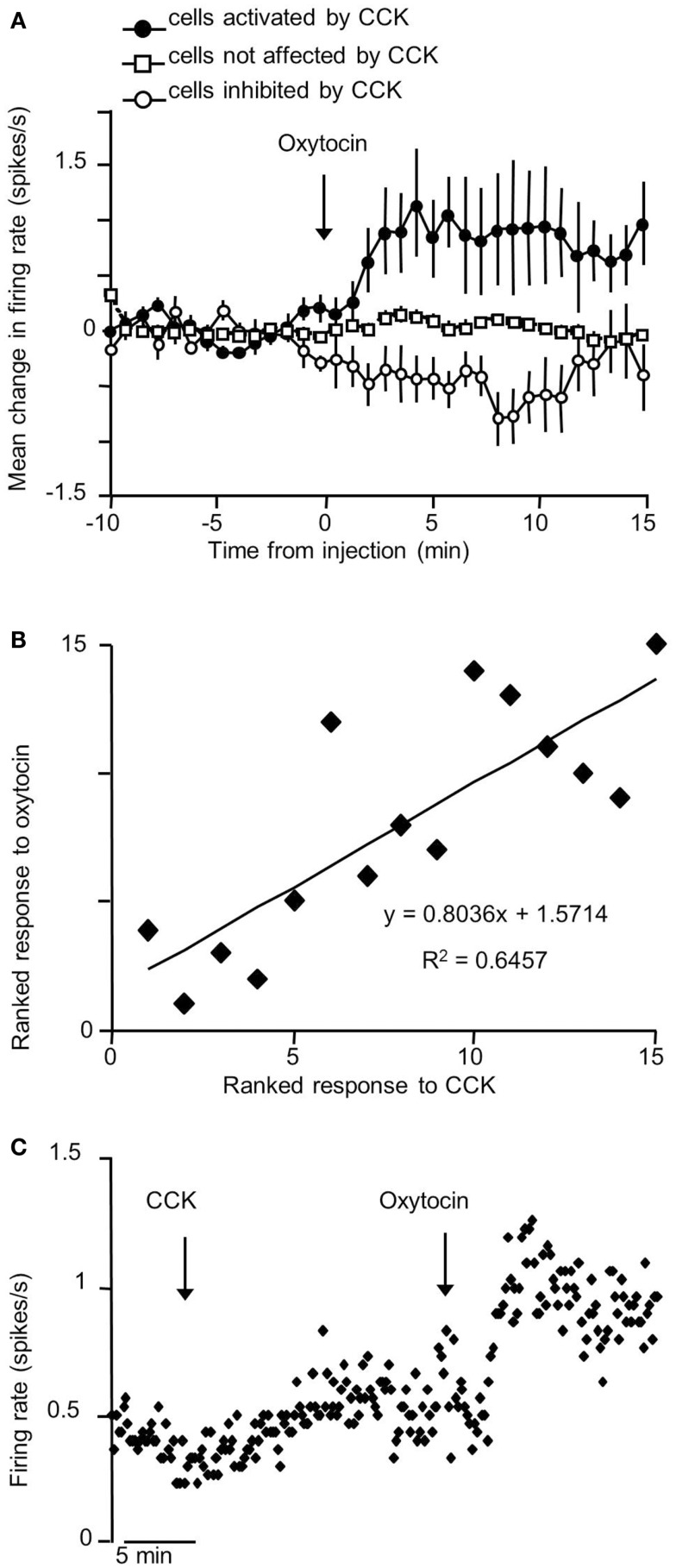
**Correlation of responses to icv oxytocin and iv CCK in VMN neurons *in vivo***. **(A)** Mean change in firing rate (±SE) in response to icv oxytocin (1–10 ng) in single VMN cells that were activated 
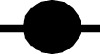
; *n* = 5), inhibited 
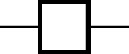
; *n* = 4), and not affected 
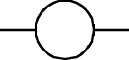
; *n* = 6) by iv CCK injection. **(B)** Rank correlation of responses to CCK and oxytocin (high ranks excited; low ranks inhibited). The solid line is the linear fit to the ranks (*r*^2^ = 0.65). **(C)** Example of convergent responses to icv oxytocin (1–10 ng) and iv CCK (20 μg/kg) in a single VMN neuron.

The VMN is not a functionally homogeneous nucleus so it is not surprising that systemic injections of CCK have diverse effects on VMN neurons, but the most common effect of CCKon the electrical activity of VMN neurons is inhibitory (Sabatier and Leng, 2010); these responses varied particularly between different subpopulations of VMN that displayed contrasting electrophysiological features. To test whether the appetite-inhibiting effects of oxytocin and those of CCK converge at the level of the VMN, we have studied the effects of central icv injection of oxytocin on the electrical activity of VMN neurons *in vivo*, and compared these responses with those of the same neurons to CCK. As with CCK, the responses to oxytocin were diverse. About 30% of neurons responded to oxytocin, 78% with a significant increase in their mean firing rate (Figure 1), and again there were differences in the responses of electrophysiologically distinct subpopulations. However, when we compared the responses of the same neurons to CCK and oxytocin, we noted particularly that, while one subpopulation of neurons had clearly divergent responses to CCK and oxytocin, in all other subpopulations the responses were remarkably convergent – for most neuronal types in the VMN, the response to icv oxytocin was a very strong predictor of the response to systemic CCK (Figure 2), supporting the hypothesis that VMN neurons are part of a common pathway mediating satiety.

**Figure 3:**
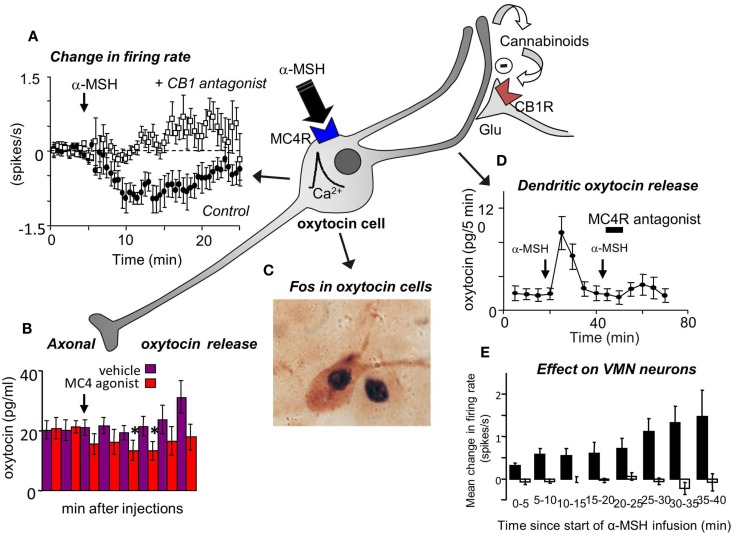
**(A)** Magnocellular oxytocin neurons are inhibited by icv injections of α-MSH and this effect can be blocked by pre-administration of a CB1 cannabinoid antagonist (Sabatier et al., 2003). **(B)** Oxytocin secretion from the posterior pituitary gland is reduced by icv injection of MC4 agonists. **(C)** However, α-MSH and MC4 agonists induce Fos expression in magnocellular oxytocin neurons and **(D)** induce dendritic oxytocin release. **(E)** Application of α-MSH retrodialysis to the SON affects the electrical activity of VMN neurons *in vivo*. Mean change in firing rate (±SE) in VMN neurons that were not affected (open bars; *n* = 13), and activated (solid bars; *n* = 8; *P* < 0.005, 0–30 min vs. control) in the 30 min period from the start of α-MSH infusion (100 μM) on the SON.

Finally, to test the feasibility of the hypothesis that oxytocin affects VMN neurons via dendritic release of oxytocin from magnocellular neurons, we studied the electrical activity of single VMN neurons while applying α-MSH directly to the ipsilateral SON by microdialysis to evoke dendritic oxytocin release. Like the effects of icv oxytocin, this too had long-lasting, mainly excitatory effects upon a subset of VMN neurons (Figure 3).

## Oxytocin and Reward

In addition to the drive to eat for metabolizable energy and nutritional factors (homeostatic feeding), humans and many other animals attend selectively to palatable foods and are motivated to eat them for pleasure (hedonic feeding). In addition to its effects on homeostatic food intake, oxytocin may have a role in the modulation of hedonic food intake.

Activation of dopaminergic mesolimbic neurons of the ventral tegmental area (VTA) is currently thought to be closely associated with reward and motivation. During palatable food intake, dopamine is released in the VTA’s target regions such as the nucleus accumbens (NAcc) and dopamine release in these structures can also be evoked by stimuli associated with or predictive of palatable food. It is likely that activation of this system equates with the motivation to attend to, pursue and consume rewarding stimuli like palatable food. Oxytocin-containing axons from the PVN contact mesolimbic neurons (Sofroniew, 1980; Succu et al., 2008) and likely have an inhibitory action as exogenous oxytocin activates NO synthase in mesolimbic dopaminergic neurons *in vivo* (Succu et al., 2008. Exogenous oxytocin also reduces amphetamine-evoked dopamine turnover in the NAcc (Qi et al., 2008) and inhibits the formation of a place-preference to methamphetamine when given into the NAcc core (Baracz et al., 2012). However, administration of oxytocin does not always lead to a reduction in dopamine release, administration into the ventral subiculum for example, a hippocampal region innervated by the PVN and potentially involved in restraining the stress response and reinstating non-food reward responding behavior, results in increased dopamine release in the NAcc (Melis et al., 2009). Inhibition of the PVN by optical stimulation of GABAergic fibers from arcuate NPY/AgRP/GABA neurons increases motivation to obtain sugar in a progressive ratio lever pressing task (Atasoy et al., 2012) suggesting that signals arising from the PVN may suppress the motivation for rewarding foods.

Consumption of readily catabolizable sugars seems to be innate in animals. Without training, rodents given a choice between water and sucrose will consume large volumes of a 10% sucrose solution [over 200 ml a day in adult male Sprague-Dawley rats (unpublished observations)] and voluntarily cease water drinking. Even transgenic mice lacking functional sweet taste receptors prefer sucrose over water (de Araujo et al., 2008) indicating that a post-ingestive reward is provided by sucrose. Transgenic mice lacking oxytocin show an enhanced preference for and consumption of sweet solutions over water in a two-bottle choice paradigm (Amico et al., 2005; Billings et al., 2006). The increase in intake is driven by a greater number of feeding bouts rather than by an increase in their duration (Sclafani et al., 2007). However, no such effect of oxytocin deficiency is seen on consumption of a palatable high-fat liquid formulation (Miedlar et al., 2007) or of a sucrose-containing solid food (Amico et al., 2005), though scheduled feeding of a high-sugar diet to rats increases oxytocin gene expression (Olszewski et al., 2009). Progressive ratio operant conditioning paradigms are often used as a measure of motivation to work for a reward. In contrast to an effect of oxytocin deficiency on freely available sweet solutions, there is less evidence that oxytocin-deficient mice have enhancements (or deficiencies) in motivation to work for food as they are not different to wild type mice in this task (Sclafani et al., 2007). In addition to Fos expression observed at the termination of bland food intake (Johnstone et al., 2006), Fos expression is also increased in PVN oxytocin neurons at the termination of a bout of feeding where only either sucrose or a high-fat food was available (Olszewski et al., 2010). In this study, hypothalamic oxytocin mRNA levels were higher in mice allowed access to a high-sugar diet compared to a high-fat diet despite both being palatable and readily consumed. Furthermore, oxytocin receptor antagonism led to an increase in sucrose consumption but has no effect on fat consumption when presented separately. Regular exposure in rats to a diet high in sugar reduces Fos expression in PVN and SON oxytocin neurons after a high- or low-sugar meal compared to animals regularly receiving a low-sugar diet (Mitra et al., 2010). This suggests that regular sugar consumption might blunt the activity of oxytocin neurons in response to any meal, whether high in sugar or not. Nonetheless, it may be that oxytocin has a satiating effect related to certain components of diet rather than a general effect. Thus, the balance of evidence suggests that oxytocin might have a role in limiting the intake of palatable food by suppressing the activation of the reward pathway.

## Conclusion

It now seems clear that oxytocin has a physiological role in energy balance through its actions in the caudal brainstem, but probably also through actions within the hypothalamus, including at the VMN, and possibly at other sites in the brain. While the actions in the brainstem appear to be part of a suite of autonomic regulatory functions exercised by parvocellular oxytocin neurons of the PVN, the hypothalamic actions appear to be more associated with motivational drive to eat, and are probably attributable to the magnocellular oxytocin system rather than the parvocellular system. It is possible that the inhibitory effects of oxytocin on appetite are closely linked to the stimulatory effects of oxytocin in sexual behavior and that this link reflects an evolutionarily conserved reciprocal regulation of these two key motivational drives (Caquineau et al., 2012). Whether it is possible to find a way of dissociating the appetite-inhibiting effects of oxytocin from the sexual arousal effects is unclear at present, but this may be the key to success in using the oxytocin system as a target for appetite-reducing drug therapies.

## Conflict of Interest Statement

The authors declare that the research was conducted in the absence of any commercial or financial relationships that could be construed as a potential conflict of interest.
